# Prevalence and associated factors of stress and anxiety among female employees of hawassa industrial park in sidama regional state, Ethiopia

**DOI:** 10.1186/s12888-023-04575-5

**Published:** 2023-02-11

**Authors:** Etenesh Kefelew, Adane Hailu, Mesfin Kote, Awgchew Teshome, Firehiwot Dawite, Mesfin Abebe

**Affiliations:** 1 School of Public Health, College of Medicine and Health Science, Arba Minch University, Arba Minch, Ethiopia; 2 Dore Bafano Primary Hospital, Sidama, Ethiopia; 3 Department of Midwifery, College of Medicine and Health Science, Dilla University, Dilla, Ethiopia

**Keywords:** Prevalence, Stress, Anxiety, Associated factors, Female, Hawassa industrial park

## Abstract

**Background:**

Work-related stress and anxiety are emerging global public health problems causing serious social and economic consequences. Working women bear a heavy burden due to high social disparity, gender inequality, and an important responsibility to balance work and family life in undeveloped society.

**Objective:**

To assess the prevalence and associated factors of work related stress and anxiety among female employees of Hawassa industrial park in Sidama Region, Ethiopia, 2021.

**Methods:**

Institution-based cross-sectional study design was conducted among 417 female employees using structured interviewer-administered questionnaires and depression, Anxiety, and Stress scale (DASS) 21 items. A simple random sampling technique was used through the computer-generated random method. The outcome variables were work related stress and anxiety. Work related stress and anxiety were ascertained using the DASS 21( stress ≥ 15 &anxiety8 – 14). The associated factors assessed included sociodemographic, behavioral factor, job and organization related factors, past illness and social support related factors. Bivariate and multivariable logistic regression analyses were done. The strength of association was declared by using an adjusted odds ratio (AOR) with a 95% confidence interval and, the statistical significance of *P-*value < 0.05.

**Result:**

The prevalence of work-related stress and anxiety were 59.3% [95% CI: (54.7, 63.9)] and 79.8% [95% CI: 75.5, 83.6)] respectively. Respondents with single marital status [AOR = 5.31, 95% CI: (1.68, 16.86)], having chronic illness [AOR = 4:00, 95% CI: (1.24, 12.9)], and current alcohol drinking [AOR = 12.5, 95% CI: (4.56, 34.2)] were significantly associated with stress. Likewise, being single in marital status [AOR = 1.99, 95% CI: (1.15, 3.46)], poor social support [AOR = 3.78, 95% CI: (1.53, 9.35)], overtime work [AOR = 2.31, 95% CI: (1.12, 4.74)], having work experience (3–4 years) [AOR = 4.71, 95% CI: (1.49, 14.84)], and fear of losing job [AOR = 1.72, 95% CI: (1.01, 2.93)] were significantly associated with anxiety.

**Conclusion:**

The prevalence of work-related stress and anxiety was high in the study area. Marital status, alcohol drinking, and chronic illnesses were factors associated with work-related stress. In contrast the fear of losing a job, work experience, overtime work, and having poor social support were factors associated with anxiety.. The significant factors identified in this study can be targeted to reduce the occurrence of work related stress and anxiety among women through designing preventive programs and strategies which includes acknowledging the importance of mental health services for the welfare of the public, screening for work related stress and anxiety, counselling, and the provision of support for women as well as lifestyle modification.

## Introduction

Work-related stress and anxiety is a growing public health problem that could result in serious but preventable social and economic consequences [1]. It affects the mental and physical health of an individual and the effectiveness of an organization [1]. World Health Organization (WHO) defines Work-related stress as people's reaction when presented with work demands and pressures that are mismatched with their knowledge that challenges their ability to cope [2]. In recent decades, progress in globalization and technology change the world working environment, introducing new forms of work organization, working relations and even employment patterns and this may lead to an increase in work-related stress and its associated disorders [3]. Work-related stress (WRS) and psychosocial problems are major problems in occupational health, involving substantial costs for staff, employers, and the government. The total number of working days lost due to stress, depression or anxiety was 11.3 million, an average of 23 days per case of stress, depression or anxiety in UK [4]. The suicide rate is also higher among individuals with WRS, 17% of suicides in Victoria Australia were work-related [5]. Moreover, WRS and anxiety can lead to a substantial decrease in employee performance to the success of the organization, affecting social, enjoyment, work interactions, and academic attainment, and leading to adverse health outcomes including death by suicide [1].

Currently, the global burden of disease and the number of people suffering from stress-related conditions caused by work is increasing. [4, 6]. High prevalence of work-related stress and psychological hazards reported in developed regions of the world. In America, according to a national survey, more than 12–16% *of* workers reported having stress, 9–13% feeling sad or depressed, or 13–19% losing sleep [7]. While In Europe, 22% of the workforce had work-related stress [6]. A cross sectional study conducted among textile manufacturing employees in Democratic Republic of Congo revealed 28% prevalence of work-related stress[8]. Furthermore, a study assessed the magnitude of work-related stress in academic institution of Tanzania found 76% of respondents had work-related stress among those 26%, 39% and 36% had high, moderate and low stress levels[9]. In Ethiopia, a study conducted in Bahir Dar city textile factory showed a high prevalence of work-related stress, 45.2% [8].

Work-related anxiety is a reaction either in terms of physiological, emotional, cognitive or behavioral reaction to some aspects of work content, work organization, and work environment. Anxiety disorder is a vague, subjective, as well as non-specific feeling of uneasiness, apprehension, tension, fears, a sense of impending doom, irrational avoidance of objects or situations, and anxiety attacks [9]. Varies factors are found to affect the prevalence of work-related stress and anxiety. Workplace conflict, prolonged working hours, low job satisfaction, and sleep problems had increased risk of anxiety and stress [10]. Furthermore, socio-professional factors, demographic factors, and Substance use such as chewing khat, drinking alcohol, and cigarette smoking are the major factors that affect stress and mental health conditions [11, 12].

Men and women are subject to different stresssors and they manifest different stress symptoms and the way they respond to manage stress is also different [13]. Women carry a double burden when they are employed in addition to paid work; women are also largely signified among unpaid contributing family workers, such as cooking, cleaning, and caring for children. Balancing responsibilities for paid and unpaid work often leads to stress, and anxiety [12]. Apart from this, Women workers in particular have disadvantaged life and working conditions due to high social disparity, gender inequality, and a great responsibility to balance work and family life in undeveloped and antisocial-democratic society [14]. It is, therefore, crucial to prevent job stress, as much as possible, before it causes chronic problems for all workers, especially women workers. they encounter different forms of mental and physical conditions, which hurt the productivity, effectiveness, psychophysical health, work ability, satisfaction, and quality of work of individual workers in the workplace [15].

Mental disorders, such as depression, anxiety and psychological distress are the leading non-communicable disorder in terms of burden in Ethiopia [16]. Evidence showed that the prevalence of common mental disorders in Ethiopia ranges from 14.9%- 27.6% in a variety of populations with higher rates among women [17, 18]. Employees who are suffering from work-related stress can lead to lower productivity, lost workdays, and a higher turnover of staff [19]. Despite this high prevalence and the huge impact of work-related stress and anxiety, there is limited evidence available in Ethiopia, regarding the prevalence and associated factors of work-related stress and anxiety among female employees in the industrial park. Therefore, this study aimed to assess the prevalence and associated factors of work-related stress and anxiety among females working in Hawassa industrial park, Sidama Region state, Ethiopia. This study will help to provide further information regarding the magnitude and factors associated with work related stress and anxiety among females to plan further interventions and to reduce its significant economic consequences on health systems and countries.

Methods and Materials.

### Study design and setting

Institution based cross-sectional study was carried out in the Hawassa industrial park from March 18 to May 18, 2021. This is found in Hawassa city. Hawassa is the capital city of the Sidama regional state. Which is located 275 km far from Addis Ababa; Ethiopia. Hawassa Industrial Park (HIP) is a nation-level textile and garment industrial park in Ethiopia. It was inaugurated in June 2016 and full operation kicked off in February 2017. It was developed and supported by the Ethiopian federal government and specialized in textile and garment production. It represents the highest level of an African textile industrial park in the perspectives of the speed of construction, size, and planning standards [15]. Currently, Hawassa industrial park contains 21 textile companies with a total of 28,948 employees among these 25,757 (89%) are female.

### Study participants

The source population was all female employees’ at Hawassa industrial park and the study population was selected female employees at selected companies of Hawassa industrial park. All women who were workers in the park for at least six months and their names listed on the salary payroll were included in the study. Those who were critically ill, and on annual or maternal leave were excluded from the study.

### Sample size determination and sampling method

The sample size was calculated by using single population proportion formula with the following assumptions: 95% level of confidence (Z = 1.96) and 5% Margin of error (d = 0.05). Proportion (*P* = 45.2%) from a study conducted in Northwest Ethiopia, 2020 [20]. Assuming a non-response rate of 10%, which gave us a total sample of 417. First, the total list of female employees in the Hawassa industrial park was taken from the salary payroll. A simple random sampling technique was used through the computer-generated random method by using Microsoft Excel to select the required sample size from the total list of employees.

### Data collection tools and procedure

Data were collected using a structured interviewer-administered questionnaire. The Questionnaires were first developed in English and then translated into the local language and back to English to check for consistency. To control the quality of the data, data were collected by trained nurses and under supervision. At enrolment, the data collector assessed socio-demographic characteristics, job and organization-related factors, past illness and social support-related factors, and behavioral characteristics (including Khat chewing, Alcohol drinking, Cigarette smoking, Coffee, Exercise and Sleep period). The Oslo-3 Social Support Scale (OSS-3) was used to assess social support-related factors: A three-item scale exploring the number of close friends, perceived level of concern from others, and perceived ease of getting help from neighbors was used to assess the level of social support..

### Measures

**Fig. 1:**
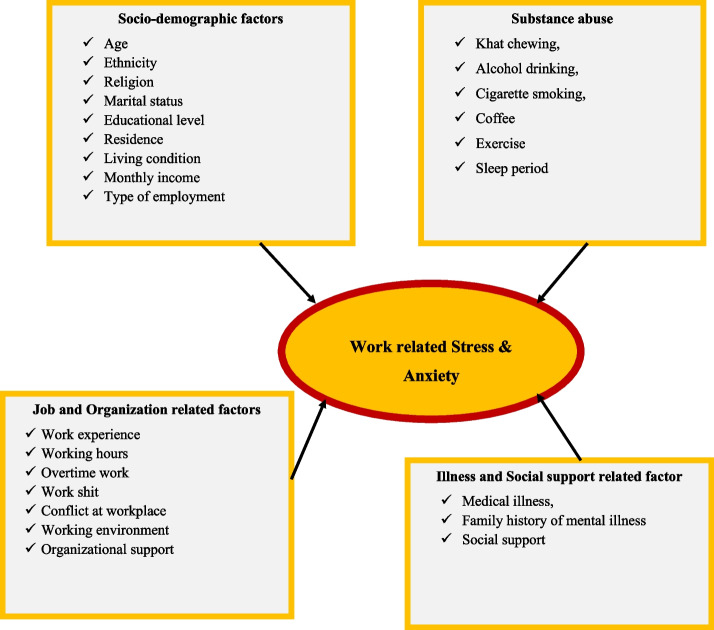
Conceptual framework for work-related stress and anxiety

Anxiety and stress were measured using Lovibond and Lovebird’s short version of the DASS-42. DASS-21 was a psychological screening instrument that is capable of differentiating symptoms of depression, anxiety, and stress. It is a validated and reliable instrument with 21 items in three domains and it is used in Ethiopia [21]. Each subscale comprises seven items assessing symptoms of depression, anxiety, and stress. In this study, only the anxiety and stress subscales were used. Participants were asked to indicate the presence of symptoms in each dimension over the past week scoring from 0 (did not apply at all) to 3 (applied most of the time). Scores from each dimension were summed. Then, the final score was multiplied by 2 and then categorized according to the DASS manual. employees who scored 15 and above on the stress subscale were considered as having stress at work [22]. Those who scored 8 and above on the anxiety subscale were considered as having anxiety. Several studies on the psychometric properties of this measure yielded consistent results [23]. The DASS 21 is a valid and useful instrument in screening stress, anxiety and depression among clinical, as well as non-clinical adult samples [24–26]. Social support:**—**was assessed by using Oslo 3-item Social Support scale. A score 3–8, 9–11, and 12–14 were considered as having poor, moderate, and strong social support, respectively [20]. Physical exercise:- in this study individuals who did work out or walked for 30 min for at least 3 days a week was considered as a performer of physical exercise [27]. The Job Content Questionnaire (JCQ) [28], and the National Institute for Occupational Safety and Health (NIOSH) generic questionnaires [29], inquired about organizational and job content related factors (overtime work, working hours, organizational support, time pressure, and physical environment). Poor organizational support is the summed scores of less than 7. High time pressure is the summed scores of more than 10. The uncomfortable physical environment is the summed score of below 9. These instruments were used in a previous study conducted among bahirdar textile factory and dukem shoe factory employees in Ethiopia and it was valid and reliable [8, 30]. The explanatory variables examined in this study included sociodemographic, job and organization related factors, past illness and social support-related factors and behavioral characteristics were explanatory variables as shown in the conceptual framework in Fig. 1.

### Statistical analysis

Data were imported to SPSS version 25 for further management and analysis. Descriptive statistics were used to describe the distribution of explanatory variables. Binary logistic regression was done and variables that showed association at the bivariate level with p-values of less than 0.25 were considered using multivariable logistic regression analysis with backward elimination. Finally, a statistically significant association was declared at a *p*-value of < 0.05. The strength of the association between the dependent variable and the explanatory variables was declared using an adjusted odds ratio at a 95%-confidence interval. Finally, the goodness of fit of the statistical model was checked using the Hosmer-Lemen show test. Model fitness was tested by the Hosmer–Lemeshow test (*P* > 0.05) which indicates a well-fitted model. For both models, the *P* value was 0.71 and 0.83 which confirmed model fitness. Multicollinearity among the independent variables in the multiple regressions was tested by estimating the Variance Inflation Factor (VIF). A VIF ≥ 10 strongly indicates Multicollinearity. For both multiple regression models, the estimated VIF for all variables ranged between 1.06 –2.10 which confirmed the absence of Multicollinearity among variables. The findings were presented in narration, tables, and figures by using proportion, median and interquartile range for data summarization.

## Results

### Characteristics of the study participants by work-related stress and anxiety

A total of 396 female employees participated in this study with a response rate of 95%. The mean age was 27.6 ± 3.5 years. The prevalence of stress was 235(59.3%), the prevalence of stress was higher in younger age (24 years), unmarried women. urban residents, those who live with Relatives, among operator, and those with a lower level of education and protestant reported higher stress than Muslims. In addition, those who drink coffee, those who have contractual employment, and those who fear the loss of their job, among those who have poor social support (68.3%vs4.4%)**(**Table 1).

**Table 1:** Characteristics of the respondent by stress and anxiety

Characteristics		Total	Stress		Anxiety	
			Yes	No	Yes	No
Overall prevalence			(59.3%)		(79.8%)	
Religion	Protestant	279(70.4)	186(66.6)	93(33.4)	216(77.4)	63(22.5)
	Orthodox	94(23.7)	68(72.3)	26(27.6)	73(77.7)	21(22.3)
	Muslim	23(5.8)	10(43.5)	13(56.5)	12(52.2)	11(47.8)
Age	≤ 24	243(61.4)	151(62.1)	92(37.9)	190(78.2)	53(21.8)
	25 – 29	103(26.0)	57(55.3)	46(44.7)	82(79.6)	21(20.4)
	≥ 30	50(12.6)	27(54)	23(46)	44(88)	6(22)
Educational Status	Read and write	22(5.6)	8(36.4)	14(63.6)	13(59.1)	9(40.9)
	Primary (1–8)	122(30.8)	67(54.9)	55(45.1)	110(90.1)	12(9.9)
	Secondary (9–12)	122(30.8)	62(50.8)	60(49.2)	47(38.5)	75(61.5)
	College and above	130(32.8)	51(39.2)	79(60.8)	58(44.6)	72(55.4)
Marital status	Single	276(69.7))	172(62.3)	104(37.7)	230(83.3)	46(16.7)
	Married	120(30.3)	63(52.5)	57(47.5)	86(71.7)	34(28.3)
Residence	Urban	249(62.9)	144(57.8)	105(42.2)	199(79.9)	50(20.1)
	Rural	147(37.1)	91(61.9)	56(38.1)	117(79.6)	30(20.4)
Current living condition	With family	137(34.6)	83(60.5)	54(39.4)	116	21
	With relative	249(62.9)	148(59.4)	101(40.6)	190(76.3)	59(23.7)
	Alone	10(2.5)	4(40)	6(60)	10(100)	-
Organizational position	Coordinator	90(22.7)	46(51.1)	44(48.9)	69(76.7)	21(23.3)
	Operator	254(64.1)	151(59.4)	103(40.9)	203(79.9)	51(20.1)
	Other	52(13.1)	37(71.2)	15(28.8)	35(67.3)	17(32.7)
Monthly income (ETB)	≤ 1500	127 (32.1)	89(70.1)	38(29.9)	105(82.7)	22(17.3)
	1500 – 3000	195 (49.2)	103(52.8)	92(47.2)	150(76.9)	45(23.1)
	> 3000	74 (18.7)	43(58.1)	31(41.9)	61(82.4)	13(17.6)
Ever chewed khat	Yes	40 (10.1)	28(70)	12(30)	40(100)	-
	No	356 (89.9)	207(58.1)	149(41.9)	80(23)	276(77)
Ever smoked cigarette	Yes	20 (5.1)	14(70)	6(30)	20(100)	-
	No	276 (94.9)	121(43.8)	155(56.2)	71(25.7)	205(74.3)
Current alcohol drinker	Yes	57(49.1)	47(82.4)	10(17.5)	22(38.6)	35(61.4)
	No	59(50.9)	19(32.2)	40(67.8)	29(49.2)	30(50.8)
drinking coffee	Yes	262 (86.1)	159(60.7)	103(39.3)	216(82.4)	46(17.5)
	No	134(13.9)	76(56.7)	58(43.3)	100(74.6)	34(25.4)
physical exercise	Yes	93 (23.5)	40(43)	53(56.9)	83(89.2)	10(10.8)
	No	303 (76.5)	195(64.4)	108(35.6)	233(76.9)	70(23.1)
Hours slept at night	< 6 h	52 (13.1)	39(75)	13(25)	21(40.4)	31(59.6)
	≥ 6 h	344(86.9)	196(57)	148(43)	98(28.5)	246(71.5)
Type of employment	Permanent	160 (40.4)	98(61.3)	62(38.8)	39(24.4)	121(75.6)
	Contractual	236 (59.6)	137(58)	99(41.9)	177(75)	59(25)
Work experience	≤ 2 years	243 (61.4)	152(62.6)	91(37.4)	186(76.5)	57(23.5)
	3–4 years	131 (33.1)	75(57.3)	56(42.7)	115(87.8)	16(12.2)
	≥ 5 years	22 (5.6)	9(40.9)	13(59.1)	15(68.2)	7(31.8)
Overtime work	Yes	90 (22.7)	76(84.5)	14(15.5	79(87.7)	11(12.2)
	No	306 (77.3)	159(51.9)	147(48.1)	237(77.4)	66(21.6)
Shift work	Yes	249 (62.9)	143(57.4)	106(42.6)	198(79.5)	51(20.5)
	No	147 (37.1)	92(62.6)	55(37.4)	118(80.3)	29(19.7)
Time pressure to finish job	Yes	347 (87.6)	204(58.8)	143(41.2)	276(79.5)	71(20.5)
	No	49 (12.4)	31(63.2)	18(36.8)	40(81.6)	9(18.4)
Conflict at workplace	Yes	308 (77.8)	186(60.4)	122(39.6)	249(80.8)	59(19.2)
	No	88 (22.2)	49(55.7)	39(44.3)	67(76.1)	21(23.9)
Working environment	Comfortable	125 (31.6)	67(53.6)	58(46.4)	95(76)	30(24)
	Uncomfortable	271(68.4)	168(62)	103(38)	221(81.5)	50(18.5)
Organizational support	Yes	174 (43.9)	120(68.9)	54(31.1)	144(82.8)	30(17.2)
	No	222 (56.1)	115(51.8)	107(48.2)	172(77.5)	50(22.5)
Fear of losing job	Yes	259 (65.4)	167(64.5)	92(35.5)	219(84.6)	40(15.4)
	No	137 (34.6)	68(49.6)	69(50.4)	97(70.8)	40(29.2)
comorbidity	Yes	92 (23.2)	77(83.7)	15(16.3)	69(75)	23(25)
	No	304 (76.8)	158(52)	146(48)	247(81.3)	57(18.8)
Family history of mental illness	Yes	78 (19.7)	42(53.8)	36(46.2)	66(84.6)	12(15.4)
	No	318 (80.3)	193(60.7)	125(39.3)	250(78.6)	68(21.4)
Social support	Poor	296 (74.7)	183(61.8)	113(38.2)	249(84.1)	47(15.9)
	Moderate	69 (17.4)	39(56.5)	30(43.5)	47(68.1)	22(31.9)
	Strong	31 (7.8)	13(41.9)	18(58.1)	20(64.5)	11(35.5)

The prevalence of anxiety was 316(79.8%); the prevalence of anxiety was higher among followers of protestant religion in younger age (24 years), unmarried women., urban residents, those who live with Relatives, among operators, and those with lower level of education and among those who drink coffee, those who do not do physical exercise, among those who feel company supports you, In addition, those who feel the working environment uncomfortable, those who fear that loss of your job, among those who have poor social support (Table 1).

### Prevalence of work-related stress and anxiety

**Fig. 2:**
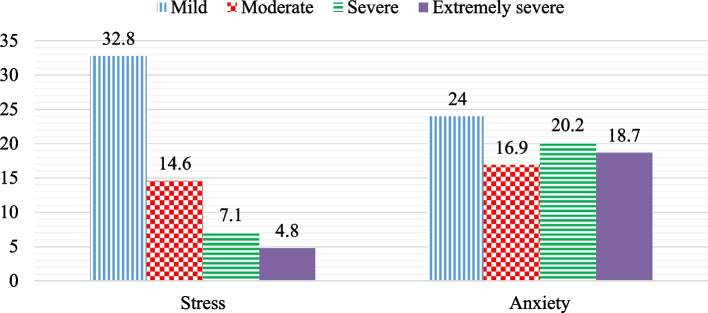
Magnitude of work-related stress and anxiety of the study participants at Hawassa Industrial Park, Sidama, Region state, Ethiopia 2021

Among the study participants, the overall prevalence of work-related stress and anxiety were found to be 59.3% [95% CI: (54.7, 63.9)] and 79.8% [95% CI: 75.5, 83.6)] respectively (Fig. 2).

Factors associated with work-related stress and anxiety among female employees.

The factors associated with stress are displayed in Table 2. The unadjusted logistic model showed that marital status, organization position, work experiences, current alcohol drinking, length of night sleep, job loss fear, social support, Coffee, and having medical illness were significantly associated with work-related stress. However, with multiple logistic regression analysis only marital status, current alcohol drinking, and having medical illness were found to be independent predictors of work-related stress. Single females had 5.31 times higher odds of work-related stress as compared to married females. [AOR = 5.31, 95% CI: (1.68, 16.86)]. The odds of having work-related stress were 12.5 times higher among current alcohol drinkers as compared to their non-drinker counterparts [AOR = 12.5, 95% CI: (4.56, 34.21)]. Moreover, female employees who have chronic medical illness had 4.00 times increased odds of work-related stress than those females who didn’t have medical illness [AOR = 4.00, 95% CI: (1.24, 12.90)].

**Table 2:** Bivariable and multivariable analysis of factors associated with stress among female employees of Hawassa Industrial Park (n = 396)

**Variables**	**Category**	**Stress**		**COR [95%CI]**	**AOR [95%CI]**
		**Yes (%)**	**No (%)**		
**Marital status**	Single	172(73.2)	104(64.6)	1.54(0.998–2.37)*	5.31(1.68–16.86) **
	Married	63(26.8)	57(35.4)	1	1
**Level of social support**	Poor	183 (77.8)	113(70.2)	2.24(1.058–4.75) *	0.273(0.011–6.84)
	Moderate	39 (16.6)	30(18.6)	1.80(0.764–4.24) *	0.104(0.003–4.299)
	Strong	13(5.5)	18(11.2)	1	1
**Current alcohol drinker**	Yes	47(71.2)	10(20)	9.89(4.13–23.72) *	12.5(4.56–34.21) **
	No	19(28.8)	40(80)	1	1
**Medical illness**	Yes	77(83.7)	15 (16.3)	4.74(2.61–8.62) *	4.00(1.24–12.90) **
	No	158(52)	146(48)	1	1
**Length of night sleep**	Less than six	39(12)	13(8.1)	2.26(1.17–4.396) *	0.35(0.081–1.536)
	Greater/equal to six	196(83.4)	148(91.9)	1	1
**Work experience**	≤ 2 years	152(64.7)	91(56.5)	2.92(1.181–7.24) *	4.121(0.309–54.96)
	3–4 years	75(31.9)	56(34.8)	2.34(0.920–5.97) *	2.022(0.129–31.76)
	≥ 5 years	8(3.4)	14(8.7)	1	1
**Fear of job loss**	Yes	167(64.5)	92(35.5)	1.84(1.21–2.81) *	2.13(0.682–6.624)
	No	68(49.6)	69(50.4)	1	
**Coffee**	Yes	159(60.7)	103(39.3)	1.18(0.84–2.648) *	0.21(0.022–2.04)
	No	76(56.7)	58(43.3)	1	1
**Organizational position**	Coordinator	46(51.1)	44(48.9)	0.42(0.11–0.64) *	3.26(0.496–21.375)
	Operator	151(59.4)	103(40.9)	0.59(0.15–0.73) *	1.00(167–6.013)
	Other	37(71.2)	15(28.8)	1	1

^Note: * Significant at *P*−value≤0.25; **Significant at *P*−Value <0.05. *COR* Crude Odds Ratio, *AOR* Adjusted Odds Ratio^

The unadjusted logistic model showed that educational status, marital status, work experience, exercise, overtime work, work environment, fear of losing a job, age, and level of social support were significantly associated with anxiety. However, after adjusting for potential confounding variables by running multivariable logistic regression work experience, marital status, level of social support, overtime work, and fear of losing a job were found to be independent predictors of work-related anxiety. The odds of having anxiety were 1.99 times higher among single females as compared to their married counterparts [AOR = 1.99, 95% CI: (1.15, 3.46)]. Moreover, female employees who fear they might lose their job had 1.72 times increased odds of anxiety as compared to their counterparts [AOR = 1.72, 95% CI: (1.01, 2.93)]. The odds of having anxiety were 3.78 times higher among females who have poor social support as compared to a female who has strong social support [AOR = 3.78, 95% CI: (1.53, 9.35)]. The odds of having anxiety were 2.31 times higher among female who works overtime in contrast to a female who does not work overtime [AOR = 2.31, 95% CI: (1.12, 4.74)]. The odds of having anxiety were 4.71 times higher among females who have work experience of (3–4 years) as compared to experienced females (≥ 5 years) [AOR = 4.71, 95% CI: (1.49, 14.84)] (Table 3).

**Table 3:** Bivariable and multivariable analysis of factors associated with anxiety among female employees of Hawassa Industrial Park (n = 396)

**Variables**	**Category**	**Anxiety**		**COR [95%CI]**	**AOR [95%CI]**
		**Yes (%)**	**No (%)**		
**Marital status**	Single	230(83.3)	46(16.7)	1.98(1.19–3.29)*	1.99(1.15–3.46) **
	Married	86(71.7)	34(28.3)	1	1
**Level of social support**	Poor	249(84.1)	47(15.9)	2.91(1.31–6.58) *	3.78(1.53–9.35) **
	Moderate	47(68.1)	22(31.9)	1.18(0.48–2.87)	1.7(0.635–4.561)
	Strong	20(64.5)	11(35.5)	1	1
**Work environment**	Comfortable	95(76)	30(24)	0.72(0.43–1.19) *	0.93(0.447–1.93)
	Uncomfortable	221(81.5)	50(18.5)	1	1
**Over time work**	Yes	79(87.7)	11(12.2)	2.09(1.05–4.15) *	2.31(1.121–4.741) **
	No	237(77.4)	66(21.6)	1	1
**Work experience**	≤ 2 years	186(76.5)	57(23.5)	1.52(0.59–3.92)	1.59(0.582–4.357)
	3–4 years	115(87.8)	16(12.2)	3.35(1.19–9.48) *	4.71(1.49–14.84) **
	≥ 5 years	15(68.2)	7(31.8)	1	1
**Fear of job loss**	Yes	219(84.6)	40(15.4)	2.26(1.37–3.72) *	1.72(1.006–2.929) **
	No	97(70.8)	40(29.2)	1	
**Exercise**	Yes	83(89.2)	10(10.8)	2.49(1.23–5.06) *	1.60(0.71–3.56)
	No	233(76.9)	70(23.1)	1	1
**Age**	< 24 years	190(78.2)	53(21.8)	0.49(0.198–1.21) *	0.47(0.178–1.22)
	25–29 years	82(79.6)	21(20.4)	0.53(0.2–1.42) *	0.77(0.26–2.31))
	> 30	44(88)	6(22)	1	1
**Coffee**	Yes	216(82.4)	46(17.5)	1.59(0.97–2.64	1.38(0.78–2.44)
	No	100(74.6)	34(25.4)	1	1

## Discussion

Reducing work place stress and anxiety is one cornerstone of achieving the 2030 Agenda for Sustainable Development Goals (SDGs). Both conditions directly impact the health-related SDGs, which seek to ensure healthy lives and promote wellbeing for all ages [31]. Globally, work-related stress and anxiety is a major challenges to workers and also organizations [1]. These issues have received limited attention, especially among female workers in developing countries including Ethiopia. This study investigated the prevalence of work-related stress and anxiety and their associated factors among females working in Hawassa industrial park, Sidama Regional state, Ethiopia. The prevalence of work-related stress among the study participant was 59.3%. The finding of this study is comparable with the prevalence reported by studies conducted at Jimma town Southwest Ethiopia (58.46%) [32], Gondar University staff in North West Ethiopia (60.4%) [33], Worabe in Region state, Ethiopia (56.3%) [34], Gondar town in North West Ethiopia (58.2%) [35] and Addis Ababa (57.3%) [36]. However, the result found from the current study was higher than the prevalence report of studies conducted in Hong Kong China (41.1%) [10], India (25%) [37], Thailand (27.5%) [38], Iran (21.3%) [39], Congo (28%) [11], Jimma University staffs (28.2%) [21], Bahir Dar Ethiopia (45.2%) [8] and Dukem Ethiopia (40.4%) [30]. The difference might be due to socio-economic, cultural, and study population characteristics difference among study population and differences in data collection tools (some of those studies use WPSS or HSE or WSQ). Moreover, the current study conducted in an Industrial park focuses on export materials that might need high demand and lead to stress. On the other hand, this finding was lower compared to the prevalence reported by a studies conducted United States[40], Pakistan [41], India [42], South Africa [43], Tanzania[44] and Bahir Dar Ethiopia[45] with prevalence of 81.8%, 94%, 77%, 68.1%, 76% and 68.2% respectively. The discrepancy of the findings might be due to work environment differences, local context including perceptions and traditions, measuring tools, and individual living standards, which could have effects on findings being high.

In this study, Single females had 5.31 times higher odds of work-related stress as compared to married females. This finding was supported by studies conducted in Addis Ababa [46]. And Jimma [21]. This might be explained by the fact that those who are married are more likely to have settled and share the burden of increasing living costs and more positive health behaviors that may contribute to reduced mental distress. Also, in this particular study most married participants were older which is a protective factor in this study. The odds of having work-related stress were 12.5 times higher among current alcohol drinkers as compared to non-drinkers. Similar findings are reported from studies conducted at Bahir Dar in Northwest Ethiopia[8], the United States [47] and meta-analysis of global studies [48]. The higher occurrence of stress among drinkers could be due to the direct effect of alcohol on the brain and the psychosocial effect of alcohol on individuals with work-related stress. In addition, unmatched work performance and high demand can create unintended negative consequences which these unintended consequences are particularly important to start substance use as coping with work-related stress [47]. In this particular study, female workers who had chronic medical illnesses had four times increased odds of work-related stress than those females who didn’t have a medical illness. Similarly, Evidence from Addis Ababa study revealed respondents who reported chronic illness were more likely to report occupational stress than those without chronic illness [46]. This could be due to that the job insecurity creating uncertainty about their future leading to stress.

The current study found that the overall prevalence of anxiety among the study participants was 79.8%. The finding of this study is consistent with studies conducted among healthcare worker in Oromia Ethiopia (78%) [49]. However, the prevalence reported in this study is higher than the reports from staffs of Jimma University in Southwestern Ethiopia (19.2%) [21], healthcare workers in Region state, Ethiopia (35.6%) [50]., nurses in Ghana 54.2% [51], a pooled prevalence of Global studies among women (27%) [52], studies conducted on working women in United Kingdom (38.9%) [53], Nurses in Hong Kong China (37%) [10], healthcare workers in Nepal (37%) [54]. at industries in Japan (32.4%) [55], physicians in China (25.7%) [56],,, Turkey healthcare workers 51.6% [57],.. Therefore, the discrepancies in the prevalence could be explained by a difference in socio-economic status, work environment, and study tool.

The study also found a significant association between marital status and anxiety. The odds of having anxiety were 1.99 times higher among single females as compared to their married counterparts. Likewise, studies conducted at Mekelle in Northern Ethiopia [58], Addis Ababa [59], and Ghana [51] reported unmarried participants had higher odds of anxiety as compared to married participants. This might be explained by the fact that those who are married are more likely to have settled and share the burden of increasing living costs and more positive health behaviors that may contribute to reduced mental distress. In addition to this,they also have better social and emotional support as compared to single females.. Also, in this particular study most married participants were older which is a protective factor in this study. Moreover, female employees who fear they might lose their job had 1.72 times increased odds of anxiety as compared to their counterparts. The finding of this study is supported by the finding of studies conducted in Addis Ababa [59], Turkey [60], United States [61] and a review of global studies [62]. The possible reason for this could be an individual's expected long-term income, averaged out over a period of years, is thus lowered as a result of the insecurity. In addition, those with few savings to fall back on may be seriously compromised by the loss of income following job loss. Since income is related to health, one could therefore expect the loss of expected income that derives from insecurity to lead to impaired mental health. Social support is also found to be a strong determinant of anxiety among employees. The odds of having anxiety were 3.78 times higher among female have poor social support as compared to female who have strong social support. Studies conducted in Ethiopia [8], Thailand [38], and China [10] revealed that employees with poor and moderate social support had a significantly higher proportion of work-related anxiety as compared to employees with strong social support. This is also supported by the finding in this study shows that the majority 249(62.9%) of female’s currently live with relatives from this 190(76.3%) show symptoms of anxiety. One possible reason for this might be there may be limited communication with family members and family members were less likely to care for each other and spend time together. The odds of having anxiety were 4.71 times higher among females who have work experience of (3–4 years) as compared to experienced females (≥ 5 years). This is in line with a study conducted in Dukem town, showing that employees who had less work experience had a higher risk of developing work-related anxiety [30]. This is due to the fact that the interaction of people with machines in the first stage and getting new incur results stress and anxiety on their work. In addition to this, the odds of having anxiety were 2.31 times higher among female who works overtime in contrast to a female who does not work overtime. This is in line with a study conducted in Dukem town, showing that employees working for more than 48 working hours per week had higher odds of developing work-related anxiety [30]. This might be because overtime work prolongs high workload, interferes with leisure activities, and causes too many employees physically and mentally fatigued to perform to the best of their ability, thereby increasing levels of anxiety [63].

This study’s main strength is its contribution to estimate the burden of stress and anxiety and the associated factors specifically among female workers. One of the limitations of the study was that the nature of the study design could not establish a clear temporal relationship between significantly associated factors and work-related stress. And also, the data were collected via a structured interviewer-administered questionnaire which may lead to information bias since mental health problem is a sensitive issues. Additionally, the study was conducted an occupational setting this might leads to healthily worker survivor effect and some of the questions were about past history and which might introduce recall bias. To accelerate progress towards the achievement of SDG 3.4 target of promoting mental health and wellbeing for all by the year 2030, there is a need to acknowledges the importance of mental health services for the welfare of the public as a health policy of FMOH. 

## Conclusion

Work related stress and anxiety was prevalent among our study population.. And the study identified respondents’ marital status, current alcohol drinking history, and presence of chronic illness as factors associated with work-related stress. Correspondingly, the study found a statistically significant association between marital status, having few years of work experience, overtime work, having poor social support, fear of losing a job, and work-related anxiety. The significant factors identified in this study can be targeted to reduce the occurrence of work related stress and anxiety among women through designing preventive programs and strategies which includes acknowledging the importance of mental health services for the welfare of the public, screening for work related stress and anxiety, counselling, and the provision of support for women as well as lifestyle modification.

## Data Availability

The datasets generated and/or analyzed during the current study are not publicly available due to confidentiality but are available from the corresponding author on reasonable request.

## Abbreviations

AOR: Adjusted odds ratio
CMI: Common mental illness
COR: Crude odds ratio
DDS: Depressive disorder syndrome
GAD: Generalized anxiety disorder
HIV/AIDS: Human immune virus/Acquired immune deficiency syndrome
OCD: Obsessive convulsive-disorder
PTSD: Post traumatic stress disorder
UK: United Kingdom
UN: United Nations
US: United States
WHO: World health organization
WRS: Work-related stress
YLD: Years lived with disability
